# Underwater image quality enhancement through composition of dual-intensity images and Rayleigh-stretching

**DOI:** 10.1186/2193-1801-3-757

**Published:** 2014-12-20

**Authors:** Ahmad Shahrizan Abdul Ghani, Nor Ashidi Mat Isa

**Affiliations:** School of Electrical & Electronics Engineering, Engineering Campus, Universiti Sains Malaysia, 14300 Nibong Tebal, Penang Malaysia; Faculty of Electrical and Automation Engineering Technology, TATI University College, Jalan Panchor, 24100 Kijal, Kemaman Malaysia

**Keywords:** Underwater image processing, Contrast and color enhancement, Noise reduction, Image details

## Abstract

The quality of underwater image is poor due to the properties of water and its impurities. The properties of water cause attenuation of light travels through the water medium, resulting in low contrast, blur, inhomogeneous lighting, and color diminishing of the underwater images. This paper proposes a method of enhancing the quality of underwater image. The proposed method consists of two stages. At the first stage, the contrast correction technique is applied to the image, where the image is applied with the modified Von Kries hypothesis and stretching the image into two different intensity images at the average value with respects to Rayleigh distribution. At the second stage, the color correction technique is applied to the image where the image is first converted into hue-saturation-value (HSV) color model. The modification of the color component increases the image color performance. Qualitative and quantitative analyses indicate that the proposed method outperforms other state-of-the-art methods in terms of contrast, details, and noise reduction.

**Electronic supplementary material:**

The online version of this article (doi:10.1186/2193-1801-3-757) contains supplementary material, which is available to authorized users.

## 1 Introduction

Due to the physical properties of water and its environment, underwater image processing has received a considerable attention since last decade. Underwater activities in discovering and recognizing objects have resulted in new challenges. Consequence from these activities, significant problems have raised due to the light absorption and diffusion effects (Church et al. 2003; Shamsudin et al. 2012; Gasparini and Schettini 2003).

As light travels in water, a rapid exponential loss of light intensity occurs depending on the color spectrum wavelength (Schettini and Corchs 2012). In clear open waters, visible light is absorbed at the longest wavelengths first (Schettini and Corchs 2012). Red, the most affected, is reduced to one-third of its intensity after 1 meter and is essentially lost after a distance of 4–5 meters underwater (Schettini and Corchs 2012). Compared with other wavelengths, the blue and violet lights are absorbed last. As such, open ocean waters appear deep-blue to the human eye.

**Figure 1:**
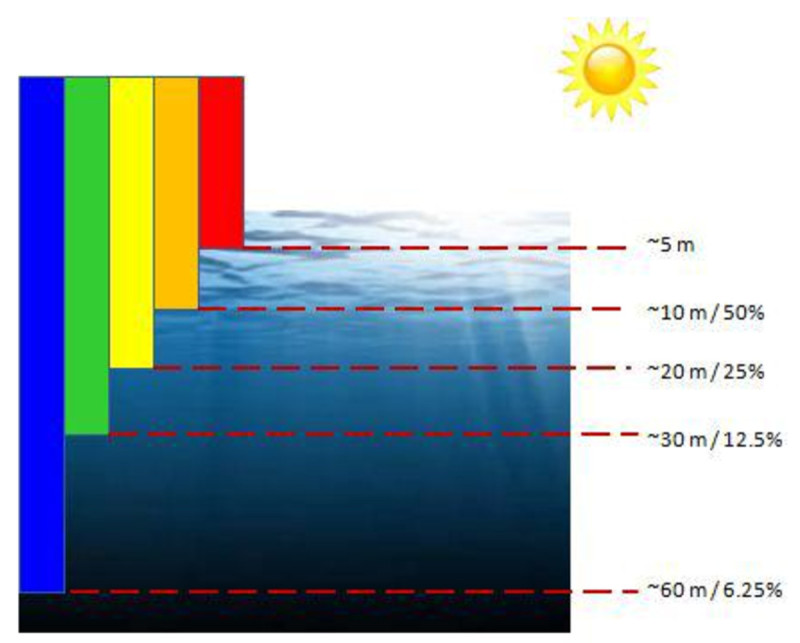
**Visual illustration of diminishing under water color (Hitam et al.** 2013 **).**

As shown in the visual illustration of diminishing under water color in Figure 1, the red color is absorbed by the water at the depth of 5 m, followed by orange, yellow, green, and blue (Hitam et al. 2013). Underwater images normally appear green-blue because these color components are last absorbed. The phenomenon of color absorption causes the captured underwater images to have low color and contrast performance. Furthermore, the important information from the image is also loss. To restore the contrast, color, and loss information from the images, the application of computer vision and image processing are gaining important.

Contrast enhancement technique is widely used for underwater image processing to improve the contrast performance. The development of the contrast enhancement technique for underwater image has attracted considerable attention in recent years. Researchers are improving the image contrast to extract as many information as possible by applying various algorithms. Numerous works have also been published based upon the models of subjective human color vision that has the widest dynamic range of color and contrast.

Bassiou and Kotropoulos (2007) have applied probability smoothing in HSI color coordinates. They demonstrate an increase in entropy and a decrease in Kullback–Leibler divergence. On the other hand, Bi-Histogram Equalization (BHE) is one of the best algorithms ever proposed to enhance the contrast of the image (Kim 1997). In this method, based on the mean value, the histogram is divided into two groups, which are independently equalized. It has been analyzed both mathematically and experimentally whereby this technique can better preserve the original brightness to a certain extent.

Rizzi et al. (2003) proposed unsupervised digital image color equalization with simultaneous global and local effects. Schechner and Karpel (2004, 2005) analyzed the physical effects of visibility degradation and proposed an image recovery algorithm based on several images taken through a polarizer at different orientations. Trucco and Olmos-Antillon (2006) devised a self-tuning image restoration filter that simplifies the well-known underwater image formation model of Jaffe (1990) and McGlamery (1979). This model proposes that an underwater image can be represented as a linear superposition of three components: direct component (light reflected directly by the object that has not been scattered in the water), forward-scattered component (light reflected by the object that has been scattered at a small angle), and back-scattered component (light reflected by the objects not on the target scene but by that enters the camera because of other reasons, such as floating particles). However, the disadvantages of physics-based methods are that they require high computing resources and consume long execution time.

Naim and Isa (2012) proposed a method called pixel distribution shifting color correction (PDSCC) for digital color image to correct the white reference point and ensure that the white reference point is achromatic. PDSCC extends the method of 3D rotational matrix (3DMAT) by implementing the modification on 2D two-color channel plane. 3DMAT is the first 3D rotational method used due to its simplicity of usage and fast execution. 3DMAT employs the 3D pixel distribution rotational by rotating the pixels three times (yaw, pitch, and roll) using three different rotational angles. In the PDSCC, two 3D rotational methods for color correction are specifically designed to shift the pixel distribution of an image such that the surrounding insignificant illumination will be suppressed or removed from the output image. This is done by moving the pixel distribution of the image to the diagonal plane of 3D RGB color model. The shifting process is implemented on 2D two-color channel plane instead of on 3D RGB color model directly. These 2D color planes are constructed from 3D RGB color model, namely red-green plane, red-blue-plane, and green-blue plane. This process could ensure the surrounding illumination pixel distribution to be rendered achromatic. From the viewer side, the method corrects the image color to become more natural. The method also intervenes with the saturation problem of the image. However, it does not optimally increase the image contrast.

Iqbal et al. proposed the integrated color model (ICM) (Iqbal et al. 2007) and the unsupervised color correction method (UCM) (Iqbal et al. 2010). In Iqbal et al. (2007), the output image in the red-green-blue (RGB) color model is stretched over the entire dynamic range. The image is then converted to the hue-saturation-intensity (HSI) color model. In this color model, the S and I components are applied with contrast stretching. After stretching, the image in HSI color model is then converted back into the RGB color model to produce an enhanced output image. In (Iqbal et al. 2010), Iqbal et al. modified two color channels, red and green, based on the Von Kries hypothesis to reduce the color cast. Contrast correction is then applied in the RGB color model. The image histograms are stretched at one or both sides based on the minimum and maximum values of each channel taken at 0.2% and 99.8% at the minimum and maximum points of the original histogram, respectively. Contrast stretching toward the upper side is applied to red, which is the lowest intensity channel. Contrast stretching at both sides is applied to green, which is the middle intensity value of the color channels, and contrast stretching at the lower side is applied to blue, which is the dominant color cast (Iqbal et al. 2010). For underwater images, the image is further converted into the HSI color model, and the S and I components are stretched at both the lower and upper sides. The overall contrast performances of the output images for both techniques increase. Nevertheless, not much difference can be observed in the output images using both techniques. However, these techniques produce high noise.

In this paper, a method of enhancing underwater image quality is proposed and explained in details. The method is proven to improve the image detail, contrast, and color while reduces the noise level by having high value of entropy and low value of MSE in comparison with the other state-of-the-art methods. The manuscript is organized as follows: In Section 1, the introduction about underwater image and the current researches progress are explained. In Section 2, the problems of underwater image are presented and the aims of this manuscript are described. Section 3 describes the proposed method in details. Qualitative and quantitative results are described in Section 4. The manuscript ends with a conclusion in Section 5.

## 2 Problem statement

Generally, underwater image processing can be addressed from two points of views. The first is image restoration technique and the second is as an image enhancement method (Schettini and Corchs 2012). Image restoration focuses on recovering a degraded image using a model of the degradation of the original image formation. These methods are rigorous and require many model parameters such as attenuation and diffusion coefficients that characterize water turbidity (Schettini and Corchs 2012). On the other hand, the image enhancement method uses qualitative and subjective criteria to produce a more visually pleasing image without the dependency on any physical model for image formation. The methods proposed by previous researchers mostly involve the modification of image pixels values that contribute towards the changes of image contrast and colors.

Therefore, in this manuscript, the proposed method is designed to use image enhancement method to enhance the underwater image contrast. This method is mostly used by previous researchers as it easier and does not involving any physical model for image enhancement. Moreover, the proposed method does not require any parameter or initial characteristic from the original image. In this proposed method, the technique is designed based on histogram modification of the original image.

**Figure 2:**
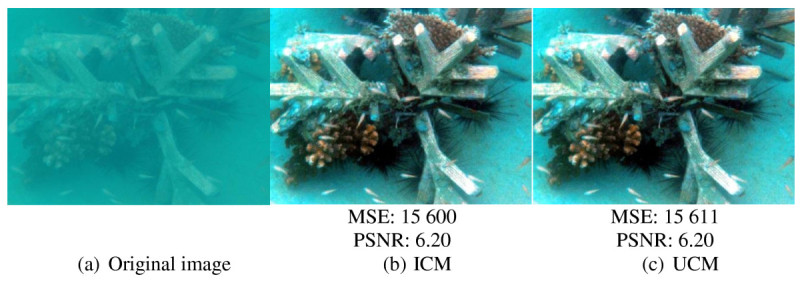
**Image of** ***Brown coral*** **. (a)** Original image, **(b)** image processed using ICM method, and **(c)** image processed using UCM method.

ICM (Iqbal et al. 2007) and UCM (Iqbal et al. 2010) are the latest methods that use the histogram modification technique to improve the quality of underwater image. ICM and UCM techniques produce a better output of underwater image in terms of contrast. Objects in the image are better recognized from the original image. However, it has a drawback as it does not significantly improve the image contrast as more blue-green illumination retains in the output image. Figure 2 shows an example of output image produced using ICM and UCM. The objects in the image are better differentiated from the background, but the blue-green illumination retains more in the output images. The color of coral is observed not significantly improved.

**Figure 3:**
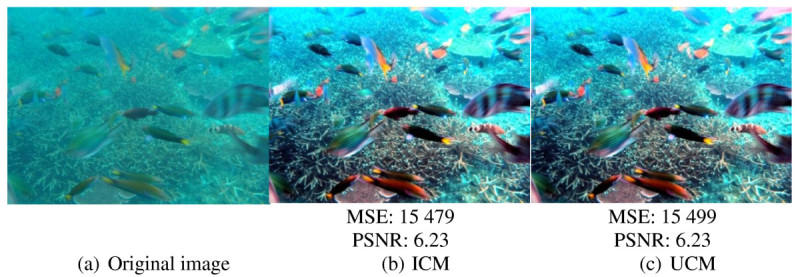
**Image of** ***Fishes*** **. (a)** Original image, **(b)** image processed using ICM method, and **(c)** image processed using UCM method.

In addition, ICM and UCM tend to produce under- and over-enhanced areas. These under- and over-enhanced areas are indicated by the darker and too bright areas in the output image. These under- and over-enhanced areas result in less information. In Figure 2, it is observed that the background objects (e.g. sand) are saturated with blue-green illumination. The same problems of under- and over-saturated areas are observed in Figure 3. The enhancement is observed more in foreground areas whereas the background areas are over-enhanced with too bright areas. Consequent from these under- and oversaturated areas, the image details at these areas are reduced.

On the other hand, ICM and UCM produce high noise in the output image resulting in changing of output image pixels. Noise is defined as the random fluctuation in the signal intensity. It is also produced from the interfering signals from electronics system of the captor device. Noise is assumed as uncorrelated with respect to the image, which means that there is no relationship between the pixels and noise values. Mean squared error (MSE) and peak signal to noise ratio (PSNR) are the quantitative metrics used to compare the improved image and original image. High noise of an image is indicated by high value of MSE and low value of PSNR. In terms of quality assessment and distortion metrics, PSNR and MSE are the most widely used (Schettini and Corchs 2012). Therefore, using the standard measurement, MSE and PSNR are used in this manuscript to measure and compare the noise between images. Referring to the images in Figures 2 and 3, the values of MSE and PSNR are stated below the images. These values show that the output images produced by ICM and UCM have high noise, showing that, there are high fluctuations in the output image intensities produced by these methods. There are also no differences or very small differences between the output images produced using these two methods.

These aforementioned problems in the output images produced by ICM and UCM are addressed in this proposed method. With the aim of improving the image contrast, increasing image details, and reducing the image noise, the proposed technique extends the use of histogram stretching.

From the results which are described in details in section 4, the output images show an improvement in terms of contrast and color. The image noise is also reduced. Moreover, the objects details in the image are also improved. The detail of the proposed method is explained in details in the next section.

## 3 Dual-intensity images and Stretched-Rayleigh distribution

This paper aims to improve the quality of underwater image by increasing the contrast, details, and visibility in underwater image. The proposed method also tends to reduce the noise in the output image as comparison to the images produced by ICM and UCM, which is mentioned previously. As the proposed method applies the histogram modification technique which is identical to the ICM and UCM, the comparison of the results will be focused between these methods.

**Figure 4:**
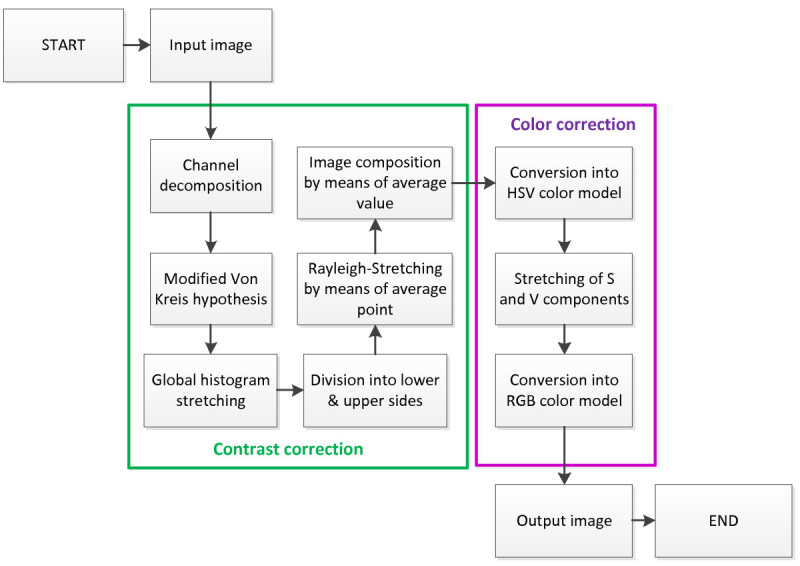
**Overall process of the proposed method.**

The proposed method applies the idea of histogram stretching in different manner from that used in the ICM and the UCM. The proposed image enhancement method is based on two main steps: first step is the contrast correction and the second step is the color correction. Figure 4 shows the process of the proposed method. In the contrast correction step, the modified Von Kries theory (Iqbal et al. 2010) is applied to each channel after the channel decomposition. Then, the global histogram stretching is applied to the image histogram. After that, the stretched histogram is divided into lower and upper sides based on the average value of corresponding color channel. After the division, the lower and upper sides of the histogram are independently stretched with respect to Rayleigh distribution to the entire dynamic range. Division and stretching processes will produce two different histograms. The images produced from these processes will have two different intensities: one is under-saturated image with low intensity value and another one is over-saturated image with high intensity value. These images are then combined by means of average value to produce an output image which has better contrast.

In the second step, the image is further processed by increasing the color performance, where the image is first converted into hue-saturation-value (HSV) color model. In the HSV color model, the S and the V components are stretched over the whole dynamic range of [0,255]. As the final step, the image is converted back into RGB color model.

The idea of dividing the image into two different intensity images is obtained by considering the bright and dark areas appear after the image processing especially by stretching the histogram. Underwater image usually consists of bright and dark areas. Overall image contrast can be increased by the global stretching of the image. The contrast of the image needs to be improved to increase the brightness of darker areas. However, global stretching also leads to an increase in the brightness of the bright areas, resulting in over-enhancement of the bright areas. These over-enhanced areas cause image pixels to become too bright, resulting in loss of image details. The same problem occurs when global stretching is applied to the darker areas. Applying global stretching to low-intensity image produces under-enhanced areas as the global stretching tends to shift the low-intensity pixel values of the image towards the smaller intensity values of the dynamic range. Thus, low-intensity pixel values of the image will have smaller intensity values which results in producing under-enhanced areas. These under-enhanced areas results in loss of image details.

### 3.1 Modified of Von Kries hypothesis

After the decomposition, image channels are applied with modified Von Kries hypothesis. Originally, Von Kries hypothesis keeps the dominant color cast channel constant (Iqbal et al. 2010). Based on this color cast, the two gain factors are calculated and multiplied with the other two color channels in order to balance the color channel correctly (Iqbal et al. 2010). However, by using the dominant color cast as the reference channel, the lowest intensity channel will have to multiply with a bigger number of multiplier. This bigger number of multiplier could lead to wrong interpretation of image color, where the object’s color in the image is deviated from their original color. Therefore, instead of using the highest intensity color channel, the proposed method used the median intensity color channel as reference channel. Hence, multipliers are not too big from the reference value and the image color will be not false interpreted. The calculated gain factors are also based on this median value.

First of all, the average values of each channel, *R*_*avg*_, *G*_*avg*_, *B*_*avg*_ are calculated (Iqbal et al. 2010; Barnard et al. 2002):

1 $$R_{avg}=\frac{1}{M\times N}\sum_{i=1}^{M}\sum_{j=1}^{N}I_{R}\left(i,j\right)$$

2 $$G_{avg}=\frac{1}{M\times N}\sum_{i=1}^{M}\sum_{j=1}^{N}I_{G}\left(i,j\right)$$

3 $$B_{avg}=\frac{1}{M\times N}\sum_{i=1}^{M}\sum_{j=1}^{N}I_{B}\left(i,j\right)$$

*M x N* indicates the number of pixels in a channel and *I*_*X*_*(i,j)* is the pixel value of respective channel at position of *(i,j)*.

The median value is determined from these three average values and used as the reference or target value. The remaining color channels are determined with multipliers (*A* and *B*) in order to produce a balanced image. The equation (4) and (5) are used to calculate the multipliers for maximum and minimum average values, respectively.

4 $$A=\frac{\mathrm{median}\left(R_{avg},\;G_{avg},B_{avg}\;\right)}{\min\left(R_{avg},\;G_{avg},B_{avg}\;\right)}$$

5 $$B=\frac{\mathrm{median}\left(R_{avg},\;G_{avg},B_{avg}\;\right)}{\max\left(R_{avg},\;G_{avg},B_{avg}\;\right)}$$

Based on the calculation in equations (1)–(3), the color channel with a minimum intensity value is multiplied with multiplier *A* whereas the color channel with a maximum intensity value is multiplied with multiplier *B*.

After multiplication with these multipliers, image channels will have balanced intensity values. As the next process, image channels will be stretched. This will be explained in detail in the next subtopic.

### 3.2 Global histogram stretching

In order to spread the pixels values of the image, the histogram of the image channels are applied with global stretching, where the histogram are stretched over the whole dynamic range of [0, 255]. This is also as preparation of the next step where the histogram will be divided into two regions based on its average value. The stretched-histogram will provide a better pixel distribution of the image channels and thus gives a more accurate average value of the channel which represents the average value of the channel for the whole dynamic range.

The equation (6) is used to stretch the histogram of respective color channel to the whole dynamic range.

6 $$P_{\mathrm{out}}=\left(P_{\mathrm{in}}-i_{\min}\right)\left(\frac{o_{\max}-o_{\min}}{i_{\max}-i_{\min}}\right)+o_{\min}\;\text{,}$$

*P*_*in*_ and *P*_*out*_ are the input and output pixels, respectively, and *i*_*min*_, *i*_*max*_, *o*_*min*_, and *o*_*max*_ are the minimum and maximum intensity level values for the input and output images, respectively.

### 3.3 Division and stretching of image histogram with respect to Rayleigh distribution

After applying the global stretching to the image histograms, the average value of each histogram will be calculated. The formulas (1) – (3) are used to determine the average values of the image channels. In this step, the value of *I*_*X*_*(i,j)* is refer to the pixel value of the new stretched-histogram which are obtained after the global stretching.

The histogram is then divided into two regions based on this average value. By dividing the histogram at its average point, two regions are produced namely lower and upper regions. The lower region should have the intensity range between 0 to the average value of the image histogram whereas the upper region should have the intensity range between the average value to the maximum intensity range of 255.

**Figure 5:**
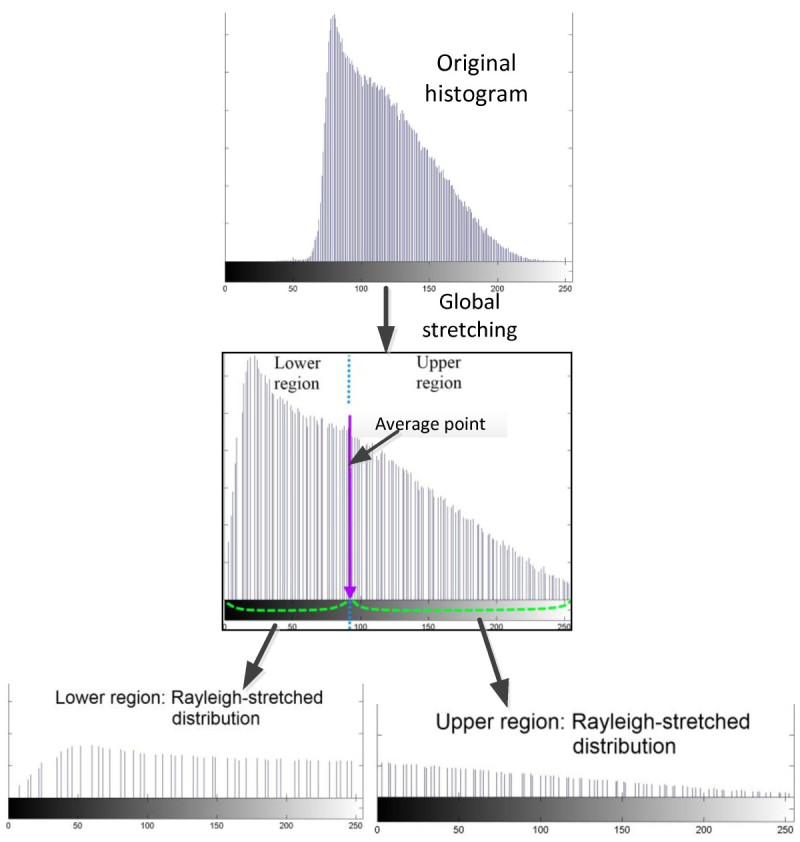
**Original histogram is applied with global stretching and divided into two separate histograms at its average point.**

For the next step, these two regions of histogram are stretched independently to produce two separated histograms. In addition to this step, these regions are stretched to follow the Rayleigh distribution over the entire dynamic range of [0, 255]. The lower region which has the intensity value between 0 to the average value will be stretched to the entire dynamic range of [0, 255]. The same process is applied to the upper region, where the initial range of the region, which lies from average value to 255 is stretched to the entire dynamic range of [0, 255]. Figure 5 shows an example of the original histogram and the histogram after the global stretching. The histogram is indicated with its average-point. This histogram is divided into two regions based on it average point and each region is stretched over the whole dynamic range with respect to Rayleigh distribution to produce two different histograms.

Rayleigh distribution is the best distribution for histogram of underwater images (Hitam et al. 2013; Eustice et al. 2002). Rayleigh distribution is refers to the bell-shaped distribution which concentrates most of the pixels at the middle of the intensity-level. The upper and lower sides of the intensity-level of the histogram will have the lowest amount of pixels. The probability distribution function of Rayleigh distribution is given by the equation (7).

7 $$PDF_{\mathrm{Rayleigh}}=\;\left(\frac{x}{\alpha^{2}}\right)e^{\left(\frac{-x^{2}}{2\alpha^{2}}\right)}\;for\;x\geq 0\;,\;\alpha >0\;\text{,}$$

where *α* is the distribution parameter of Rayleigh distribution and *x* is the input data which is, in this case, the intensity value.

In order to map the histogram to follow the Rayleigh distribution, the equation (6) is integrated with equation (7) to produce Rayleigh-stretched distribution as in equation (8).

8 $$\mathrm{Rayl}.-\mathrm{stretched}=\;\frac{\left[\left(P_{\mathrm{in}}-i_{\min}\right)\left(\frac{o_{\max}-o_{\min}}{i_{\max}-i_{\min}}\right)+o_{\min}\right]}{\alpha^{2}}\cdot e^{\frac{-{\left[\left(P_{\mathrm{in}}-i_{\min}\right)\left(\frac{o_{\max}-o_{\min}}{i_{\max}-i_{\min}}\right)+o_{\min}\right]}^{2}}{2\alpha^{2}}}$$

The output histogram is stretched over the entire dynamic range of [0, 255]. Therefore, the values of *o*_*max*_ and *o*_*min*_ can be substituted with the values of 255 and 0, respectively. Thus, equation (8) can be simplified as equation (9):

9 $$\mathrm{Rayl}.-\mathrm{stretched}=\;\frac{255\left(P_{\mathrm{in}}-i_{\min}\right)}{\alpha^{2}\left(i_{\max}-i_{\min}\right)}\cdot e^{\frac{-{\left[255\left(P_{\mathrm{in}}-i_{\min}\right)\right]}^{2}}{2\sigma^{2}{\left(i_{\max}-i_{\min}\right)}^{2}}}\;\text{,}$$

where *i*_*min*_ and *i*_*max*_ indicate the minimum and maximum intensity level values for input image in each region, respectively.

For the lower region, *i*_*min*_ indicates the minimum intensity level value of the image histogram, and *i*_*max*_ indicates the maximum intensity level value in the region, which is equivalent to the average-value of the histogram. For the upper region, *i*_*min*_ indicates the minimum intensity level value in the region which is equivalent to the average-value of the image histogram, and *i*_*max*_ indicates the maximum intensity level value in the region which is equivalent to the maximum intensity level of the image histogram. The described processes are applied to all channels of RGB color model.

### 3.4 Image composition by means of average value

In the division and stretching processes, every channel of the RGB color model will produce two different histograms: lower-stretched histogram and upper-stretched histogram. For the next step, all of these histograms are combined to produce images in RGB color model. All lower-stretched histograms from these three channels of RGB color model are composed to produce an image and all upper-stretched histograms are also composed to produce another image. This process produces two different images with different contrasts. The lower-stretched histograms produce an under-enhanced image, whereas the upper-stretched histograms produce an over-enhanced image. These two images are then composed by means of average values between these two images.

In order to compose these two images, the images are stacked together. With the z-axis as reference, the images are aligned together so that the images are located perpendicular to the z-axis and the image plane itself. The under-enhanced image is then projected with the over-enhanced image. Each pixel of both images is compared at a particular pixel location, and the average value from these pixels is determined.

**Figure 6:**
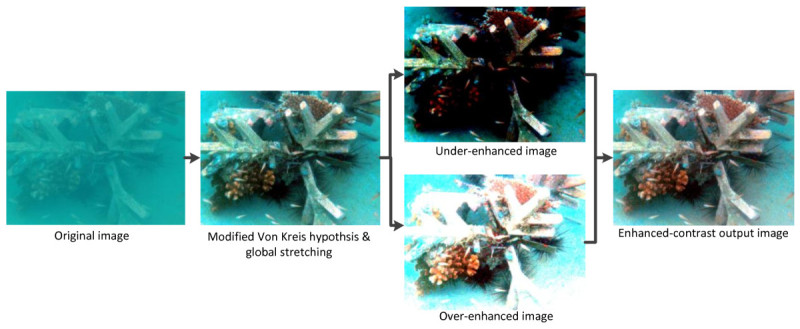
**The visual process of contrast correction of the proposed method.**

This process produces an improved image in terms of contrast, with the output image having better contrast. The resultant image has average values between the under- and over-enhanced images as well as excellent contrast and visibility. Figure 6 shows the visual contrast correction process of the proposed technique. The original image is applied with modified Von Kries hypothesis and global stretching. The image is then divided into two regions based on its average value resulting in producing two different intensities images. Finally, these images are combined by means of its average value to produce an enhanced-contrast output image.

### 3.5 Conversion into HSV color model: limited-stretching of S and V components

After the contrast correction in RGB color model, the image will undergo the color correction process. In this process, the underwater image is converted into hue-saturation-value (HSV) color model to improve the color performance. HSV color model is used to modify the image saturation and the value. Value in HSV color model is equivalent to the image brightness. Saturation and brightness in an image are the important parameters of clearness and visibility. Therefore, the objects in the image can be clearly differentiated from the background.

Firstly, the image is decomposed into respective channel. Then, the histograms of S and V components are stretched. To reduce the effects of the under- and over-saturated image, the stretching processes are limited to 1% from the lower and upper limits of the output histograms. Therefore, the stretching processes are applied to the both histograms of saturation and value at 1% to 99%.

**Figure 7:**
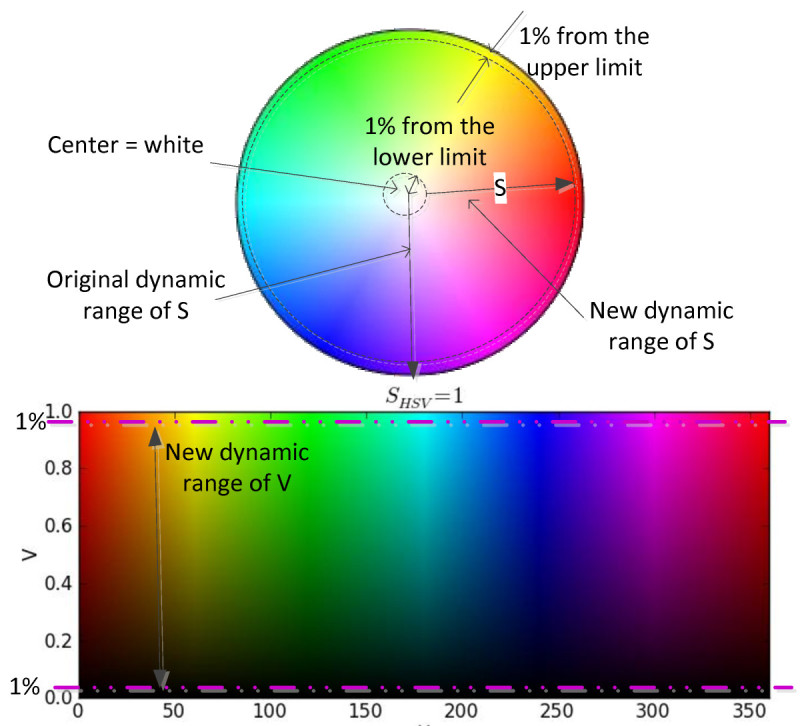
**New range of S and V components at 1% of minimum and maximum limits in HSV color model which is applied in the proposed method.**

Figure 7 shows the new limits of S and V components of HSV color model which is applied in the proposed method. The stretching process of the S and V components is limited at 1% of their minimum and maximum values.

After the stretching of S and V components, the H-S-V components are composed and the image is converted back into RGB color model.

## 4 Results and discussion

As mentioned before, the comparison of the results of the proposed method will be focused with the ICM and UCM methods as these methods are the latest methods which are most similar to the proposed method in terms of histogram modification. In addition to that, the PDSCC (Naim and Isa 2012) is also used to compare the resultant images as PDSCC is the latest technique in image processing which is also design for underwater image. Histogram equalization (HE) (Singhai and Rawat 2007; Yeganeh et al. 2008; Senthilkumaran and Thimmiaraja 2014; Arici et al. 2009) will be also used in comparison as it is the basic technique in histogram modification that most commonly used by previous researchers in image processing. The proposed method is designed to improve the underwater image in terms of contrast, noise reduction, and image details, quantitative measurement such as entropy, mean square error (MSE), and peak signal to noise ratio (PSNR) are used to evaluate the output image.

Qualitative results in section 4.1 will be used to evaluate the image contrast and partly supports the evaluation of image details. In section 4.2, quantitative results will be used to evaluate the image noise and also to support the result in section 4.1 in terms of image details. Entropy value is used to evaluate the image details whereas the image noise will be evaluated using the values of MSE and PSNR.

Nevertheless, most of the previous researchers measure the improvement of image quality through visual inspection (Schettini and Corchs 2012). A few of them use quantitative estimations and residual error which is computed between ground truths and corrected images (Schettini and Corchs 2012).

Contrast of an image is quantitatively hard to measure. Moreover, there are several more definitions proposed for contrast measurement as stated in (Rizzi et al. 2004). However, there is no reference to compare an image from another in terms of contrast. Contrast value is just a scale. Low value of contrast causes the image contrast to under-saturate whereas high value of contrast causes the image contrast to over-saturate. The optimal contrast should lie between low and high contrast values. However, there is no comparative contrast value to show that one image is better than the other image in terms of contrast. Therefore, in the proposed method, the evaluation of image contrast is done through the evaluation of human visual system (HVS). The observers will evaluate the resultants image based on their observation.

On the other hand, image noise is evaluated through the value of MSE and PSNR. Noise is defined as the random fluctuations in the signal intensity. It is produced from the interfering signals from electronics system of the captor device. Noise is assumed as uncorrelated with respect to the image, which means that there is no relationship between the pixels and noise values. In terms of quality assessment and distortion metrics, the peak signal to noise ratio (PSNR) and the mean squared error (MSE) are the most widely used (Schettini and Corchs 2012; Hitam et al. 2013; Kumar and Rattan 2012). Therefore, in the proposed method, MSE and PSNR are used as they are the most quality assessment metrics used by previous researchers and widely used in the image evaluation.

Entropy or discrete entropy is used as quality assessment in (Singhai and Rawat 2007; Wu and Li 2013; Wu et al. 2005; Ye 2009; Padmavathi et al. 2010). Entropy represents the abundance of image information. Entropy is a measure of image information content, which is interpreted as the average uncertainty of information source (Ye 2009). In an image, entropy is defined as corresponding states of gray-level which individual pixels can adopt. Information content, *I*_*i*_ of a particular gray-level is defined as equation (10), where *p(x)* is the probability distribution function of a certain gray-level.

10 $$I_{i}=-log_{2}p\left(x\right)$$

Entropy is calculated as the summation of the products of the probability of outcome multiplied by the log of the inverse of the outcome probability. As stated in (Wu et al. 2005) and (Ye 2009), the entropy in equation (11) is used to measure the image information. The performance of image details or image information in the proposed method is also measured using this formula.

11 $$H\left(X\right)\;=\;-\sum_{x=1}^{k}p\left(x\right)\log_{2}p\left(x\right)$$

*p(x)* represents the probability distribution function of the image at the state *x* (pixel). *k* is the number of gray-level. If an image is in the format of unsigned integer 8, it will be [11111111_b_] in binary number, then there will be 256_d_ (decimal) gray-level which means that the first gray-level is 0 and the last gray-level is 255. In this paper, the images used are in the format of unsigned integer 8 which means that the first gray-level (x = 1) is 0 and the last gray-level (k = 256) is 255.

To evaluate the proposed technique, underwater images which are captured in three different Malaysian islands, namely Tioman, Langkawi, and Perhentian are used in the experiments. These different underwater images are captured with different environments, objects, and backgrounds.

### 4.1 Qualitative result

**Figure 8:**
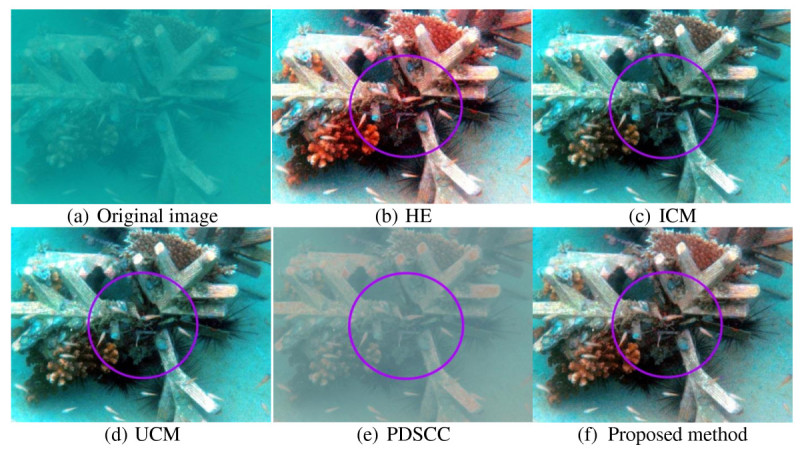
***Brown coral*** **. (a)** original image. The rest are images processed using the following methods **(b)** HE, **(c)** ICM, **(d)** UCM, **(e)** PDSCC, **(f)** Proposed method.

**Figure 9:**
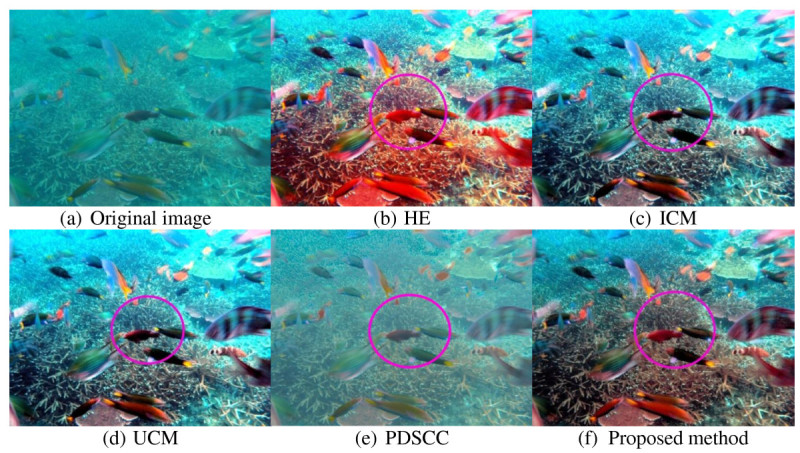
***Fishes*** **. (a)** original image. The rest are images processed using the following methods **(b)** HE; **(c)** ICM; **(d)** UCM; **(e)** PDSCC; **(f)** Proposed method.

**Figure 10:**
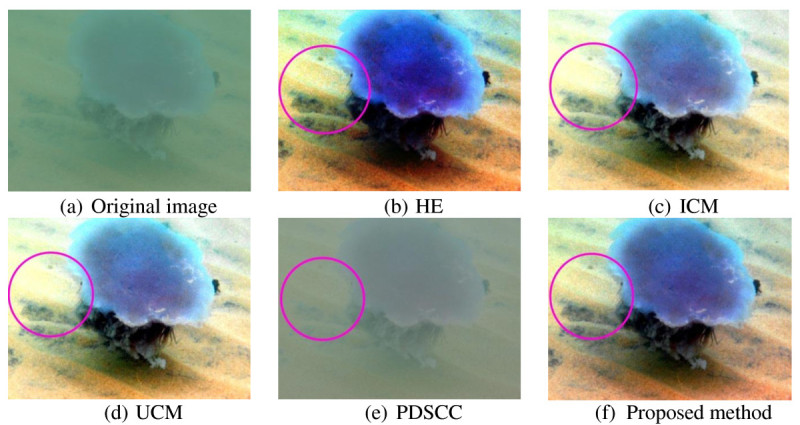
***Jellyfish*** **. (a)** original image. The rest are images processed using the following methods **(b)** HE, **(c)** ICM, **(d)** UCM, **(e)** PDSCC, **(f)** Proposed method.

**Figure 11:**
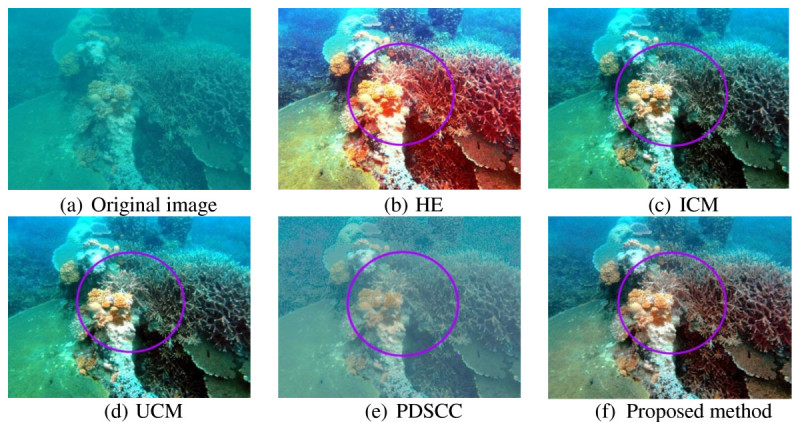
***Coral leaf*** **. (a)** original image. The rest are images processed using the following methods **(b)** HE, **(c)** ICM, **(d)** UCM, **(e)** PDSCC, **(f)** Proposed method.

Underwater images that used to evaluate the proposed method have very low contrast and visibility. The objects in the images are unclear, thus, making differentiate between the objects and the background difficult. For qualitative result, the improvement of resultant image is described in term of contrast, visibility and details. Three hundred underwater images are used to test the proposed method. Out of 300 underwater images, four underwater images are shown in this paper as examples. Figures 8, 9, 10 and 11 show examples of images processed using the proposed method in comparison with the other methods. The circled areas in each image show clear differences between these compared images.

The image of brown coral in Figure 8 shows that the contrast of the image produced by HE is over-saturated. This produces reddish and too bright areas in the output image. ICM and UCM over-saturate the image contrast as the blue-green color of the image becomes too bright. From the observation, the improvement of image contrast is limited as the image retains its blue-green illumination. Moreover, the color of coral is also not significantly improved. PDSCC improves only a small amount of contrast as the object and the background cannot be differentiated and seen clearly. It turns the blue-green illumination into reddish. The proposed method improves the image contrast better as the coral is better differentiated from the background. The object color is also better improved and less blue-green illumination retained. The circled areas clearly show the differences between these compared images.

The circled area in Figure 9 shows that the image produced by the HE is over-saturated as the image become reddish and the blue-illumination becomes too bright. The images produced by ICM and UCM are also over-saturated as the background areas in the image retain more blue-green illumination, resulting in unclear background objects. From the observation, the corals in the background areas become out of shape as there too much blue-green illumination is. PDSCC removes some blue-green illumination in the output image, but the contrast is not significantly improved because the corals and fishes cannot be differentiated clearly. The color performance of the output image is also low. Unlike the other methods, the output image produced by the proposed method has better contrast as the objects are better differentiated from the background. There is only very small amount of blue-green illumination in the background areas.

The output image of the HE in Figure 10 shows that the image become over-saturated and some areas are darker. The images produced by ICM and UCM are also over-saturated as the images become too bright and the sand is out of shape, resulting in reduction of the images details. PDSCC improves only a small amount of contrast as the jellyfish cannot be differentiated clearly from the background. The image produced by the proposed method increase the contrast as the object and the background is clearly differentiated. Moreover, the image is not saturated or over-enhanced. The circled areas in the figures clearly show the differences.

As observed in Figure 11, the image contrast produced by HE is over-saturated as the image becomes too bright and reddish. ICM and UCM do not significantly improve the images contrast as there is more blue-green illumination in the output images. Moreover, the coral in the background is also over-saturated. PDSCC reduces the blue-green illumination in the output image but the overall contrast does not improve as the objects are hardly differentiated from the background. The proposed method improves the contrast better as the objects in the image are better differentiated from the background. In addition, the blue-green illumination is reduced more compared to the other methods.

Qualitative results in Figures 8, 9, 10 and 11 show that there are significant improvement in the output images produced by the proposed method. Unlike the other methods, the proposed method increases the image contrast better as the objects in the image are better differentiated from the background. From the observation, the proposed method reduces the blue-green water effect. The object color is also better enhanced. These improvement have proved that the proposed method successfully enhance the image contrast and increase the image details of the output image.

Additional file 1 shows more qualitative results of the tested underwater images. In Additional file 2, the quantitative evaluation of the images in Additional file 1 is presented in a table. Images in Additional file 1 show that the overall image contrast and color of the proposed method are significantly improved better than other methods.

### 4.2 Quantitative result

As mentioned previously, the proposed method aims to improve the image contrast, increase the image details, and reduce the image noise in comparison with ICM and UCM as these methods are the latest and most identical to the proposed method. Image contrast is evaluated trough the visual observation in section 4.1. Image details could be also partially evaluated through the observation of the image in section 4.1. In addition, to support the evaluation of the image details, the value of entropy is used. As mentioned at the beginning of section 4, high entropy value indicates that the image is more details and has more information whereas low entropy value shows that the image has less information. The entropy of an image is given by the equation (11). To evaluate the image noise, the values of MSE and PSNR are used.

The MSE and the PSNR are two error metrics used to compare the quality of resultant images. The MSE is the cumulative squared error between the resultant and original images. It is given by the equation (12) (Hitam et al. 2013):

12 $$MSE=\frac{1}{M\times N}\;\sum_{M,N}{\left[I_{1}\left(m,n\right)-I_{2}\left(m,n\right)\right]}^{2}$$

where *I*_*1*_*(m,n)* and *I*_*2*_*(m,n)* represent the original and improved values of images pixels intensities, respectively. *M* × *N* denotes the size of the image, and *m* and *n* indicate the x and y locations of the pixels of the image.

The PSNR indicates the ratio of the maximum possible signal and the noise. It is given by the equation (13) (Hitam et al. 2013):

13 $$\mathrm{PSNR}=20Log_{10}\frac{\left(2^{B}-1\right)}{\sqrt{MSE}}\;\text{,}$$

where *B* represents the bits per sample.

The best resultant image is indicated by low MSE, high PSNR, and high entropy.

Table 1 shows the comparative values of entropy, MSE, and PSNR for the images shown in Figures 8, 9, 10 and 11. As shown in the table, the quantitative performance of the proposed method stands ahead of the other methods in terms of entropy. This proves that the resultant images of the proposed method contain the most details and information. However, in terms of MSE and PSNR, the proposed method is only second to the PDSCC. The PDSCC produces the lowest MSE and the highest PSNR for these four images. However, visual observation in section 4.1 indicates that the PDSCC cannot improve the contrast optimally as the blue-green illumination of the water remains. The objects in the image processed by the PDSCC cannot be seen clearly because of the limited difference in the object and its background. The PDSCC has low entropy, causing the resultant images to contain less detail.

**Table 1:** **Quantitative results in terms of the Entropy, MSE, and PSNR for images in Figures** 8 **,** 9 **,** 10 **and** 11

Image	Method	Entropy	MSE	PSNR
**Brown coral**	Original image	6.341	-	-
	HE	5.807	8 759	8.71
	ICM	7.486	15 600	6.20
	UCM	7.649	15 611	6.20
	PDSCC	6.360	**3 802**	**12.33**
	Proposed method	**7.866**	5 317	10.87
**Fishes**	Original image	7.341	-	-
	HE	5.935	6 806	9.80
	ICM	7.742	15 479	6.23
	UCM	7.652	15 499	6.23
	PDSCC	7.198	**1 487**	**16.41**
	Proposed method	**7.764**	3 424	12.79
**Jellyfish**	Original image	6.218	-	-
	HE	5.647	4 534	11.57
	ICM	7.479	15 207	6.31
	UCM	7.046	15 214	6.31
	PDSCC	5.623	**202**	**25.09**
	Proposed method	**7.857**	3 149	13.15
**Coral leaf**	Original image	6.902	-	-
	HE	5.795	8 000	9.10
	ICM	7.591	14 531	6.51
	UCM	7.449	14 546	6.50
	PDSCC	6.982	**1 982**	**15.28**
	Proposed method	**7.702**	3 801	12.33

Note: The values in bold typeface represent the best result obtained in the comparison.

These quantitative results prove that the proposed method successfully reduces the noise and increases the image details unlike the ICM and UCM.

The dataset in Table 2 shows the average values of entropy, MSE, and PSNR for 300 tested underwater images. The proposed method leads other methods in terms of entropy as it has the highest value of entropy. The high value of entropy shows that the image produced using the proposed method has most details and information. The proposed method ranks second in terms of the MSE and the PSNR after the PDSCC.

**Table 2:** **Average values of Entropy, MSE, and PSNR for 300 tested underwater images**

Method	Entropy	MSE	PSNR
Original image	7.001	-	-
HE	5.860	5 220	11.34
ICM	7.592	12 652	7.23
UCM	7.575	12 656	7.23
PDSCC	6.846	**936**	**20.92**
Proposed method	**7.726**	2 847	14.15

Note: The values in bold typeface represent the best results obtained in the comparison.

These values indicate a degree of improvement in terms of contrast and lowering of the noise in the resultant image produced using the proposed method. Visual inspection shows that objects in the image are clear and better differentiated than those obtained using other methods.

## 5 Conclusion

The proposed method has successfully improved the contrast, increase the details, and reduce the noise of underwater images. As mentioned previously, the proposed method focuses the comparison with ICM and UCM. Qualitative and quantitative evaluations prove that the proposed method outperforms the methods of ICM (Iqbal et al. 2007) and UCM (Iqbal et al. 2010) which are previously proposed by Iqbal et al. Combination of histogram modification in the RGB and HSV color spaces improves the image contrast and color, resulting in the increase of the visibility and details of underwater image. This method is expected could help to further the underwater research.

The overall results of the HE indicate that the image is over-saturated and become reddish, and thus image details are reduced. The ICM and UCM improve the image contrast. However, these methods produce high noise and low details. Moreover, some areas of the underwater images are over-saturated. On the other hand, the resultant images produced by the PDSCC displays excellent MSE and PSNR. However, the image contrast is not significantly increased because the blue-green illumination retains in the images. Several images are only partially increased, and only a part of water illumination is removed. Moreover, the objects and the background cannot be clearly seen and are hardly differentiated.

## Electronic supplementary material

Additional file 1: **Shows the results of 10 tested underwater images for more comparison.** The quantitative parameters for these images are presented in Additional file 2. (DOC 3 MB)

Additional file 2: The table shows the quantitative values of entropy, MSE, and PSNR for the images in Additional file 1. (DOC 64 KB)
