# Supplementary material for: Underwater image quality enhancement through composition of dual-intensity images and Rayleigh-stretching
Source: Springerplus. 2014 Dec 20;3:757. doi: 10.1186/2193-1801-3-757 (PMC4320174; doi:10.1186/2193-1801-3-757)
Supplement: Supplementary file 1 — Additional file 1: Shows the results of 10 tested underwater images for more comparison. The quantitative parameters for these images are presented in Additional file 2. (DOC 3 MB) [file 40064_2014_1510_MOESM1_ESM.doc]

**Additional file 1**

Shows the results of 10 tested underwater images for more comparison. The quantitative parameters for these images are presented in Appendix B.

| **Original** | **HE** | **ICM** | **UCM** | **PDSCC** | **Proposed method** |
| --- | --- | --- | --- | --- | --- |
| **Image 1** | | | | | |
| **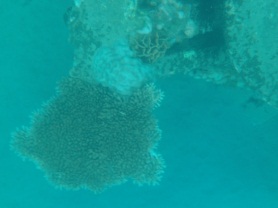** | **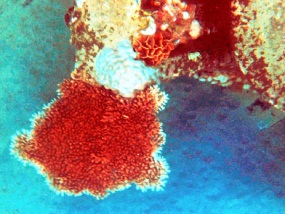** | **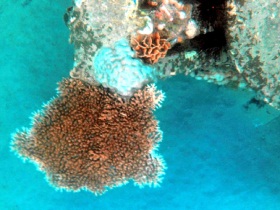** | **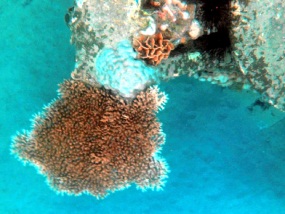** | **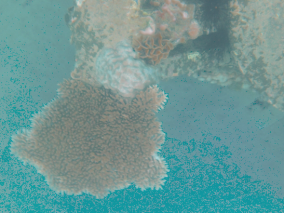** | **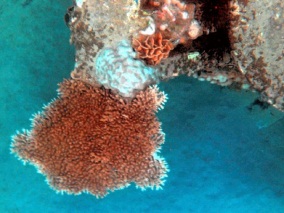** |
| **Image 2** | | | | | |
| **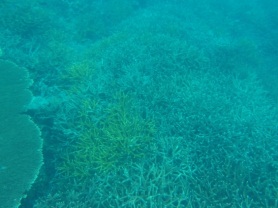** | **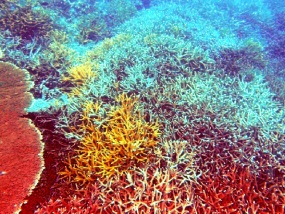** | **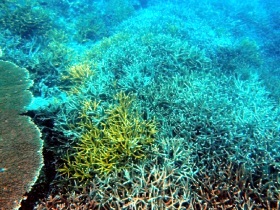** | **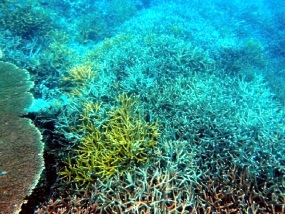** | **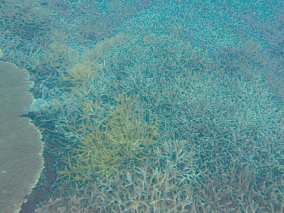** | **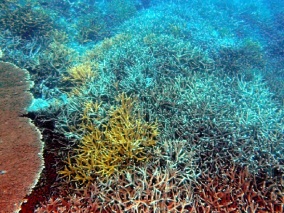** |
| **Image 3** | | | | | |
| **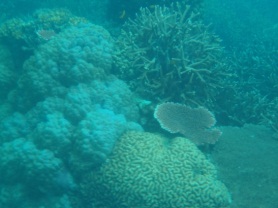** | **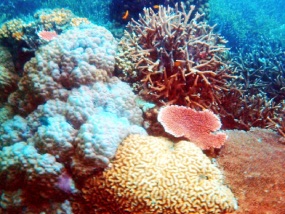** | **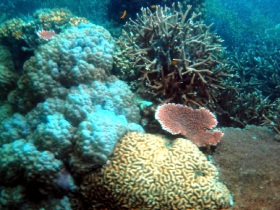** | **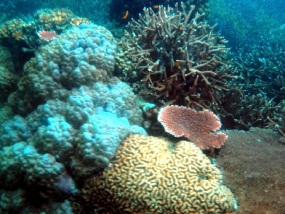** | **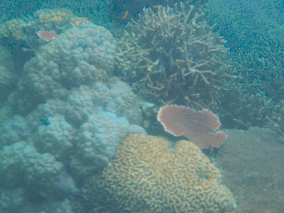** | **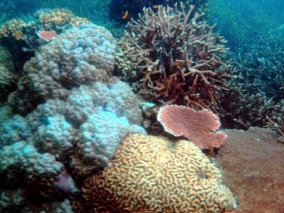** |
| **Image 4** | | | | | |
| **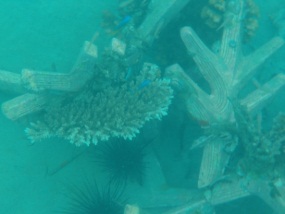** | **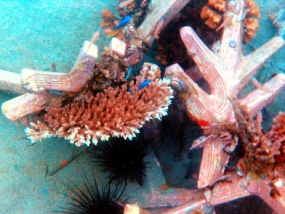** | **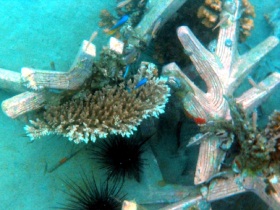** | **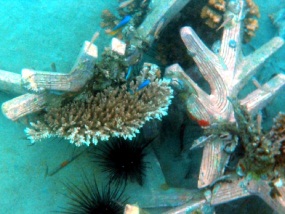** | **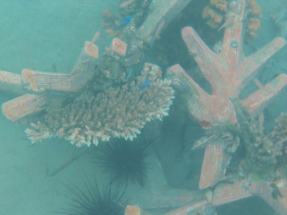** | **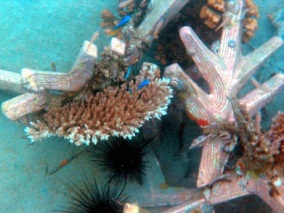** |
| **Image 5** | | | | | |
| **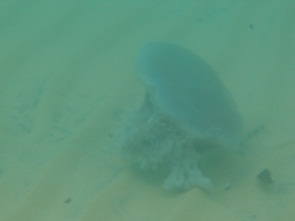** | **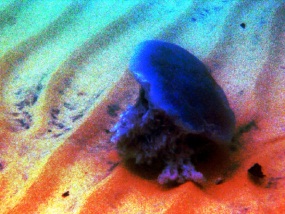** | **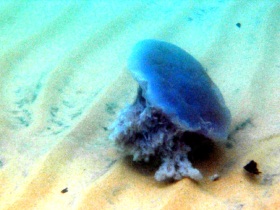** | **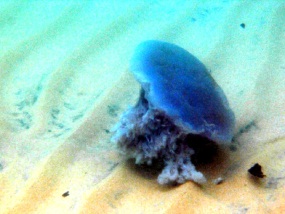** | **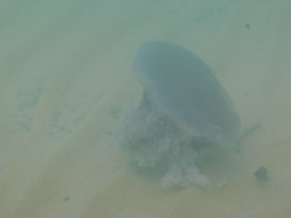** | **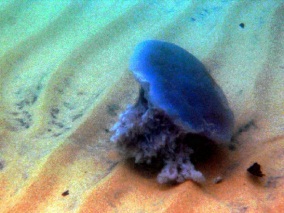** |
| **Image 6** | | | | | |
| **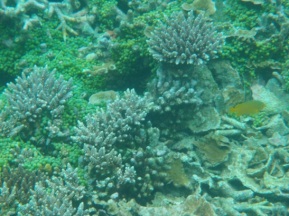** | **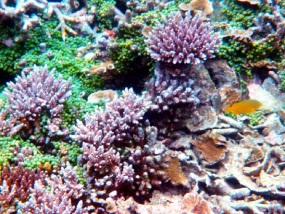** | **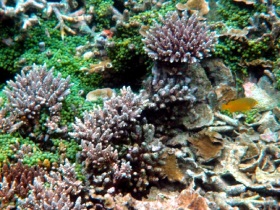** | **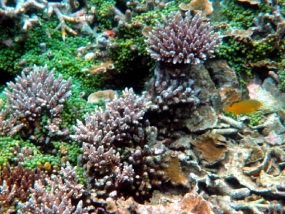** | **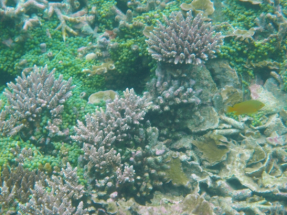** | **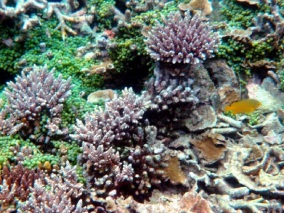** |
| **Image 7** | | | | | |
| **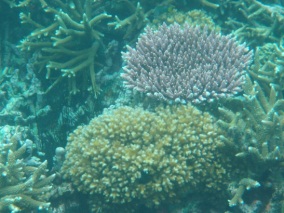** | **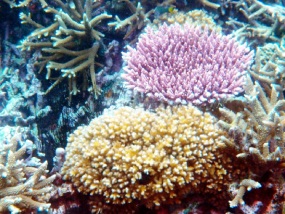** | **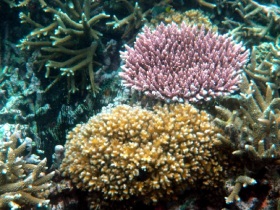** | **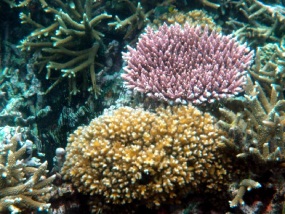** | **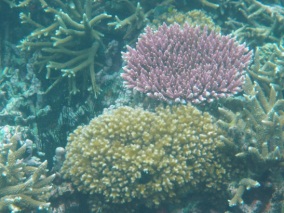** | **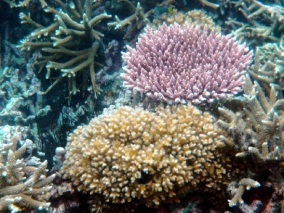** |
| **Image 8** | | | | | |
| **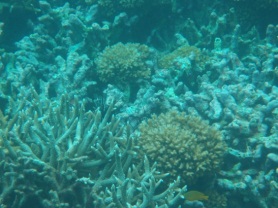** | **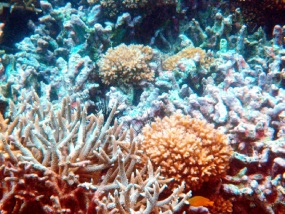** | **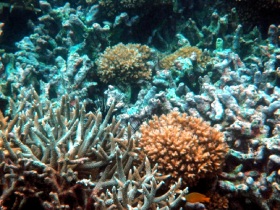** | **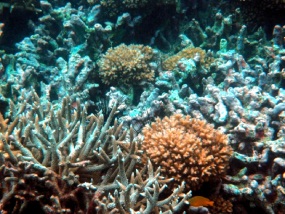** | **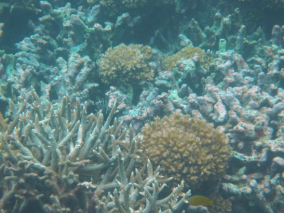** | **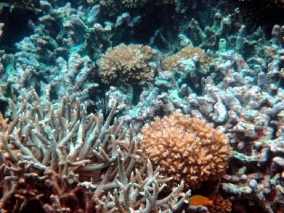** |
| **Image 9** | | | | | |
| **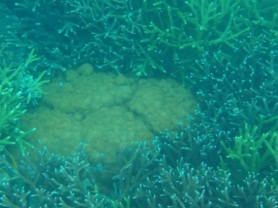** | **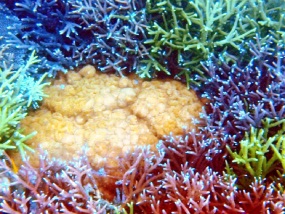** | **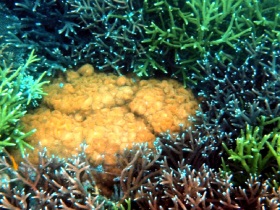** | **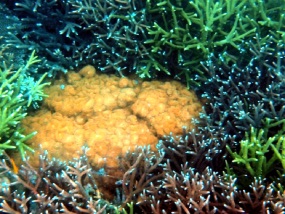** | **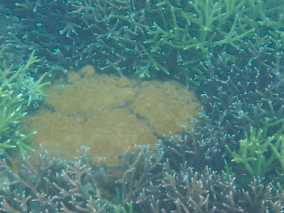** | **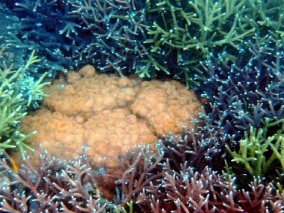** |
| **Image 10** | | | | | |
| **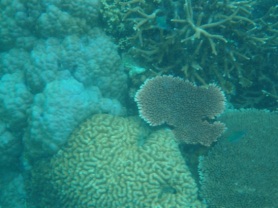** | **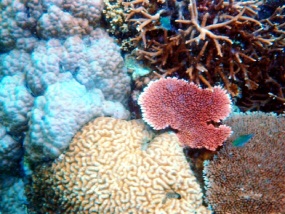** | **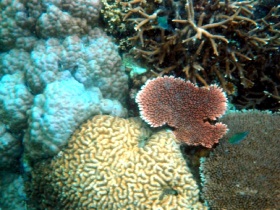** | **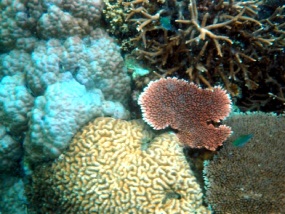** | **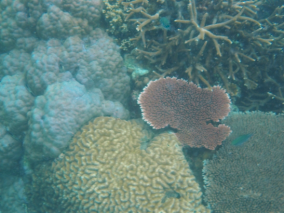** | **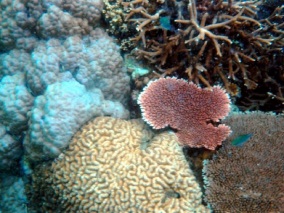** |
