# Supplementary material for: Underwater image quality enhancement through composition of dual-intensity images and Rayleigh-stretching
Source: Springerplus. 2014 Dec 20;3:757. doi: 10.1186/2193-1801-3-757 (PMC4320174; doi:10.1186/2193-1801-3-757)
Supplement: Supplementary file 2 — Additional file 2: The table shows the quantitative values of entropy, MSE, and PSNR for the images in Additional file 1. (DOC 64 KB) [file 40064_2014_1510_MOESM2_ESM.doc]

**Additional file 2**

The table shows the quantitative values of entropy, MSE, and PSNR for the images in Additional file 1.

Note: The values in bold typeface represent the best results obtained in the comparison.

| **Image** | **Method**  **Evaluation** | **HE** | **ICM** | **UCM** | **PDSCC** | **Proposed method** |
| --- | --- | --- | --- | --- | --- | --- |
| Image 1 | Entropy | 5.806 | 7.407 | 7.384 | 6.351 | **7.706** |
|  | MSE | 8 517 | 15 580 | 15 599 | **3 383** | 4 376 |
|  | PSNR | 8.83 | 6.21 | 6.20 | **12.84** | 11.72 |
| Image 2 | Entropy | 5.672 | 7.482 | 7.256 | 6.572 | **7.843** |
|  | MSE | 9 181 | 14 435 | 14 449 | **3 237** | 4 407 |
|  | PSNR | 8.50 | 6.54 | 6.53 | **13.03** | 11.69 |
| Image 3 | Entropy | 5.935 | 7.702 | 7.591 | 6.875 | **7.794** |
|  | MSE | 6 977 | 13 107 | 13 112 | **1 857** | 4 032 |
|  | PSNR | 9.69 | 6.96 | 6.95 | **15.44** | 12.08 |
| Image 4 | Entropy | 5.948 | 7.642 | 7.682 | 6.748 | **7.880** |
|  | MSE | 7 029 | 14 997 | 15 004 | **2 269** | 4 266 |
|  | PSNR | 9.66 | 6.37 | 6.37 | **14.57** | 11.83 |
| Image 5 | Entropy | 5.544 | 7.086 | 6.764 | 5.469 | **7.808** |
|  | MSE | 4 976 | 15 437 | 15 446 | **426** | 3 164 |
|  | PSNR | 11.16 | 6.25 | 6.24 | **21.84** | 13.13 |
| Image 6 | Entropy | 5.988 | 7.854 | 7.852 | 7.336 | **7.912** |
|  | MSE | 2 926 | 14 172 | 14 174 | **497** | 1 965 |
|  | PSNR | 13.47 | 6.62 | 6.62 | **21.17** | 15.20 |
| Image 7 | Entropy | 5.983 | 7.749 | 7.711 | 7.330 | **7.877** |
|  | MSE | 2 705 | 14 384 | 14 385 | **239** | 1 692 |
|  | PSNR | 13.81 | 6.55 | 6.55 | **24.35** | 12.85 |
| Image 8 | Entropy | 5.977 | 7.741 | 7.685 | 7.191 | **7.824** |
|  | MSE | 4 717 | 12 344 | 12 349 | **1 121** | 2 812 |
|  | PSNR | 11.39 | 7.22 | 7.21 | **17.63** | 13.64 |
| Image 9 | Entropy | 5.907 | 7.600 | 7.374 | 6.843 | **7.798** |
|  | MSE | 6 826 | 11 339 | 11 344 | **1 372** | 3 952 |
|  | PSNR | 9.79 | 7.59 | 7.58 | **16.76** | 12.16 |
| Image 10 | Entropy | 5.988 | 7.797 | 7.750 | 7.074 | **7.886** |
|  | MSE | 4 805 | 11 967 | 11 970 | **996** | 3 016 |
|  | PSNR | 11.22 | 7.35 | 7.35 | **18.15** | 13.34 |
